# Editorial: Exploring system justification phenomenon among disadvantaged individuals

**DOI:** 10.3389/fpsyg.2022.1104400

**Published:** 2023-01-05

**Authors:** Luca Caricati, Chuma Kevin Owuamalam, Annalisa Casini, Stefano Passini, Gianluigi Moscato

**Affiliations:** ^1^Department of Humanities, Social Sciences and Cultural Industries, University of Parma, Parma, Italy; ^2^Department of Psychology, Reed College, Portland, OR, United States; ^3^Psychological Sciences Research Institute, Université Catholique de Louvain (UCLouvain), Louvain-la-Neuve, Belgium; ^4^Department of Education Studies, University of Bologna, Bologna, Italy; ^5^Department of Social Psychology, Social Work, Social Anthropology and East Asian Studies, University of Malaga, Málaga, Spain

**Keywords:** system justification, social disadvantage, oppression, social hierarchy, intergroup conflict, social change

The question of *why* (or even when) the disadvantaged might be more or less supportive of existing social arrangements is a matter of debate amongst social and political psychologists (e.g., Passini, 2019; Jost, 2020, see also Rubin et al., 2022). Accordingly, for this Research Topic, we chose a title that was deliberately broad in scope, accommodating several aspects that included: (a) the drivers of system justification; (b) the socio-structural conditions that enhance or dampen system justification, (c) the ideological correlates of system support, and (d) the impact of system justification on wellbeing. Taken together, the contributions comprised in this Research Topic provide a comprehensive analysis of these four issues.

## The drivers of system justification

Two articles explicitly examined the motivational basis for system justification. Using a large cross-national sample of participants from 40 different nations, Caricati et al. found that trust in institutions of governance (a manifestation of system justification) increased as a positive function of (a) the degree to which citizens invested in their national identity, and (b) improvements in citizens' outcomes relative to others overtime (see also Caricati, 2018; Caricati and Owuamalam, 2020), and both these effects were visible even after controlling for national wealth and inequality. In a complementary manner, Owuamalam et al. reported results from two studies showing that support for a Brexit/Leave vote in UK's 2016 EU referendum and a Trump administration in the 2016 presidential election were mostly explained by group interest than by epistemic and existential needs (cf. Jost, 2020).

## Socio-structural aspects of system justification

The issue of how socio-structural conditions might influence the probability of system justification was tackled by three contributions. Ferrari et al. and Lönnqvis et al. highlight that difference in status does play an important role. Indeed, using a large cross-national sample of 16 European countries, Ferrari et al. found that while homonegativity was inversely related to trusting the system, gender-based social status crucially moderated this relationship, with this negative association being stronger for men (the higher-status group) than for women. A similar status-based link to system justification was also reported by Lönnqvist et al. who found a positive association between socio-economic status and system support in the Hungarian electoral context, using two representative samples of the Hungarian population surveyed in 2010 and 2018. Finally, focusing this time on the disadvantaged alone, Degner et al. used an open-ended question format to examine the reasons displayed by gay men/lesbians, African Americans, overweight people, and the elderly for explaining social inequality. Results showed that the disadvantaged rarely used system-justifying stereotypes to explain status differences. Instead, Degner et al. found indications that social reality constraints/pressures could be a powerful explanation for status differentials (see also Owuamalam et al., 2019a,b).

## Ideological correlates of system justification

Three articles considered the effect of holding ideological beliefs such as ambivalent sexism (Glick and Fiske, 1996) and social dominance orientation (Sidanius and Pratto, 1999) on system justification. Chayinska et al. investigated the relations between system justification, ambivalent sexism, and support for traditional, husband-centered marital surname change in three cross-sectional studies, with two samples of women in Turkey and one in the United States. Results consistently showed that hostile sexism, but not benevolent sexism, predicted support for marital surname change among Women: an association that was partially mediated by gender system justification. Furthermore, in two experimental studies, Carvalho et al. found that social dominance orientation increased the motivation to engage in direct competition with a relevant higher-status outgroup. Finally, Lönnqvist et al. showed that low-SES people who were strongly invested in the political ideology in power, reported stronger system justification. Their results further revealed that although levels of authoritarianism were substantially unchanged, system justification tended to increase from 2010 to 2018 in Hungary: suggesting a highly variable trend in the perceived legitimacy of the existing political system of governance.

## Wellbeing and system justification

Finally, a set of articles dealt with the connection between system justification and wellbeing. Panari and Tonelli addressed the question of “what makes the unemployed more likely to accept their disadvantaged position or oppose their situation by searching for a better job?” performing a review of the literature about protean career orientation (i.e., the extent to which individuals feel responsible for their career choices and search for self-realization; Briscoe and Hall, 2006). Results suggested that personal empowerment is key when it comes to helping people to switch from a legitimizing perception of their disadvantaged position (resulting in a lack of search for employment or acceptance of any job), to a more proactive and agentic view of their situation (resulting in a search for a job that is consistent with their life aspirations). Finally, Möller et al. investigated if and how first-generation students (lower status) and students with university-educated parents (higher status) used different defense mechanisms (e.g., university-system justification, academic identification, and social belonging) to cope with the threat of lockdown due to the COVID-19 pandemic. Results from a large sample of German-speaking students (*N* = 848) showed that system justification reduced threat appraisals, but mostly among the higher-status group. Low-status groups, however, relied on personal relations with other students as well as academic identification to cope with the COVID-19 threat.

## Concluding remarks

By taking different approaches, we believe the papers in this Research Topic provide valuable new insights into the phenomenon of system justification in general, and among disadvantaged people in particular. Of course, contributions to this volume contain various limitations that the authors themselves also identified, which makes related conclusions somewhat tentative at this time. Nevertheless, we believe these articles highlight novel areas in the literature on system justification that ought to be considered when investigating the processes of support for unequal societal systems. We hope that the present Research Topic would stimulate further discussions and help in our quest to better understand the processes of (and controversies surrounding) system-justifying attitudes amongst the disadvantaged.

## Author contributions

LC, CO, and AC drafted the manuscript and which all authors reviewed and approved for publication. All authors contributed to the article and approved the submitted version.
